# Association of maternal ultra-processed food consumption during pregnancy with atopic dermatitis in infancy: Korean Mothers and Children’s Environmental Health (MOCEH) study

**DOI:** 10.1186/s12937-024-00969-7

**Published:** 2024-06-25

**Authors:** Won Jang, Minji Kim, Eunhee Ha, Hyesook Kim

**Affiliations:** 1 Department of Food and Nutrition, Wonkwang University, Iksan, Korea; 2 Institute for Better Living, Wonkwang University, Iksan, Korea; 3 Graduate Program in System Health Science and Engineering, College of Medicine, Ewha Womans University, Seoul, Republic of Korea; 4 Department of Environmental Medicine, School of Medicine, Ewha Womans University, Seoul, Republic of Korea; 5 Institute of Ewha-SCL for Environmental Health (IESEH), College of Medicine, Ewha Womans University, Seoul, Republic of Korea; 6 Department of Medical Science, College of Medicine, Ewha Medical Research Institute, Ewha Womans University, Seoul, Republic of Korea

**Keywords:** Atopic dermatitis, Ultra-processed food, Pregnant women, Birth cohort study, Community-based collaborative network

## Abstract

**Background:**

Maternal diet during pregnancy might influence the development of childhood allergic disorders. There are few studies on the association between processed food intake and infant atopic dermatitis (AD) during pregnancy. The aim of the present study was to investigate the association of ultra-processed food (UPF) intake during pregnancy with infantile AD.

**Methods:**

This study involved 861 pairs of pregnant women and their offspring from the Mothers’ and Children’s Environmental Health (MOCEH) study, a multi-center birth cohort project conducted in Korea. Dietary intake was estimated using a 24-h recall method at 12−28 weeks gestation. The NOVA classification was used to identify UPF, and UPF intake was calculated as the percentage of total energy consumption and categorized into quartiles. Infantile AD was assessed based on medical history and the criteria of the International Study of Asthma and Allergies in Childhood (ISAAC). Associations were assessed by logistic regression with adjustment for confounding factors.

**Results:**

Children born to mothers in the highest quartile of UPF consumption (15.5% or more of the total energy) compared to the lowest quartile (6.8% or less) showed a higher risk of AD within 12 months [odds ratio (OR) = 1.69; 95% confidence interval (CI): 1.07−2.66, *P* for trend 0.0436]. After adjustment for the confounding factors under study, the association was strengthened; the adjusted OR between extreme quartiles was 2.19 (95% CI: 1.11–4.32, *P* for trend = 0.0418). This association was maintained even after an additional adjustment based on the Korean Healthy Eating Index (KHEI), an indicator of diet quality.

**Conclusions:**

Higher maternal consumption of UPF during pregnancy was associated with a greater risk of infantile AD within the first year of life.

## Background

Atopic dermatitis (AD), also referred to as eczema, is a chronic inflammatory skin condition marked by dry, red, and pruritic (itchy) skin [1, 2]. AD usually initiates in infancy and affects up to one-fifth of children [3]. The condition not only leads to a diminished quality of life but also exerts adverse effects on psychological well-being, social interactions, and physical health [4]. Hence, early prevention of atopy is crucial to mitigate these impacts.

The precise pathogenesis of AD is not fully understood, but it is believed to be influenced by a combination of environmental and genetic factors, including hygiene, gut microbial diversity, pollution, climate, and diet [5]. The health and disease origin hypothesis proposes that the prenatal period plays a pivotal role in shaping the immune function of the fetus, with a potential increased risk of allergic diseases due to exposure to detrimental factors during this time [6, 7]. Of particular note is the growing body of research focusing on the relationship between maternal dietary factors, nutrients, and the development of AD in infants [8–17]. Studies in this area seek to understand how maternal diet may influence the risk and occurrence of AD in their offspring. Previous studies have found that the dietary intake of specific foods, including fish [8–10], fruits [11, 12], vegetables [11, 12], and dairy products [13], as well as certain nutrients like antioxidant nutrients [11, 12] and polyunsaturated fatty acids [14, 15] during pregnancy, was significantly associated with a reduced risk of AD in offspring. Conversely, higher maternal intakes of meat and sodium during pregnancy may be associated with a higher risk of AD in the offspring [10, 16]. These results must be interpreted with caution, however, because the traditional approach to evaluating the nutritional effects of foods is to focus on individual foods, which can neither uncover interactions between nutrients nor detect the effects of single nutrients [17].

Instead, assessing diet by analyzing the consumption levels of ultra-processed foods (UPF), as defined by the NOVA classification proposed by Monteiro et al., offers a more comprehensive assessment of meals compared to solely considering individual food groups or nutrients [18, 19]. Under the NOVA classification, UPF is one of four groups categorized by the extent and purpose of processing [18]. Ultra-processing creates appealing, hyper-palatable, cost-effective, and convenient products. However, nutritionally, these products are often energy-dense and contain high amounts of saturated fats, refined starches, added sugar, or salt [18], and are generally associated with promoting obesity and inflammatory responses [20]. Additionally, there have been reports indicating an association between the consumption of processed foods and increased AD prevalence [21]. Maternal UPF consumption during pregnancy may have adverse health effects on the offspring, including increased risks of obesity [22] and cognitive impairment [23, 24].

To our knowledge, there is no study on the association between maternal UPF consumption and infantile AD. Therefore, the purpose of the present study was to explore the association of maternal UPF consumption during pregnancy with infantile AD in a Korean population.

## Methods

### Study participants

**Fig. 1:**
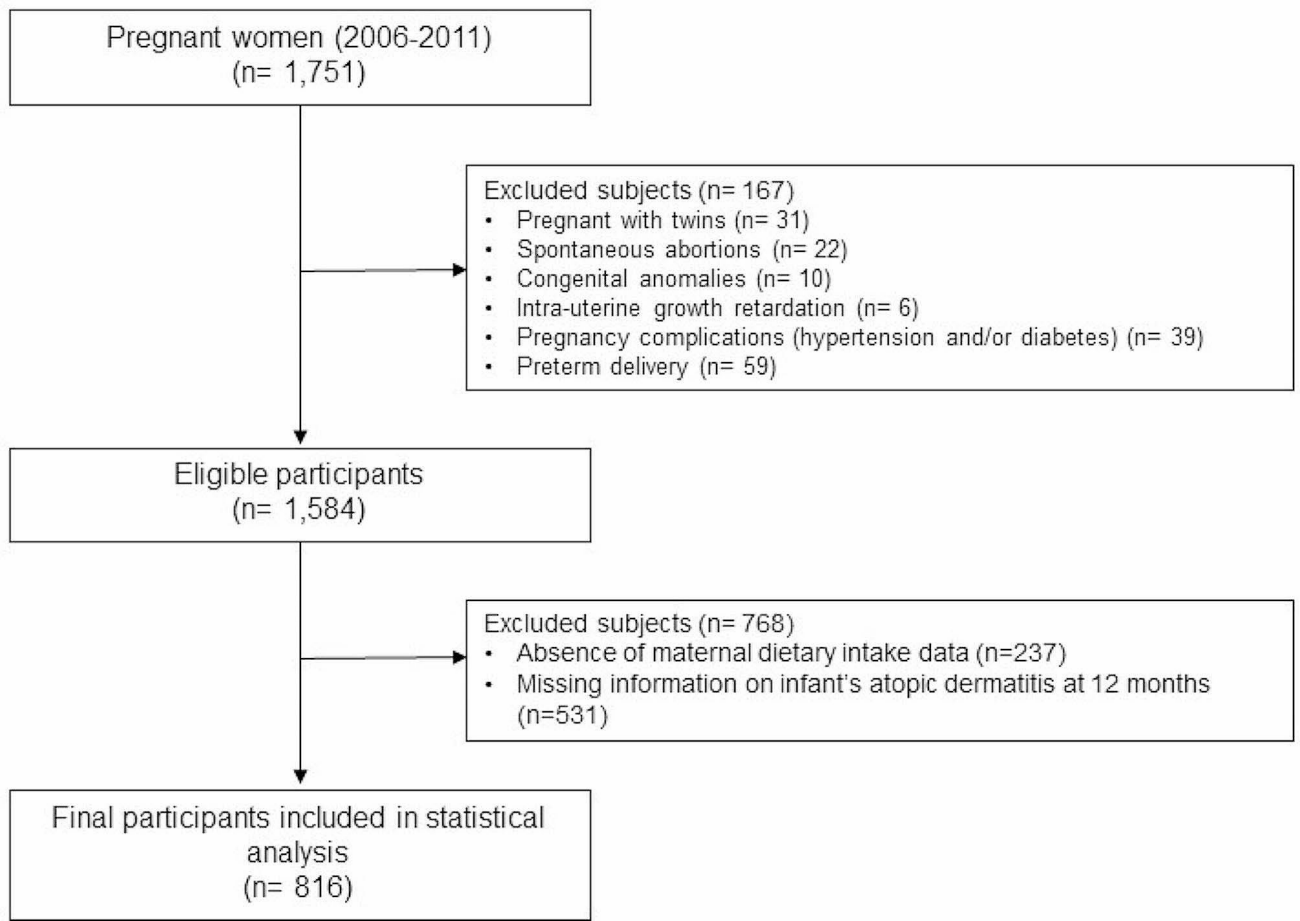
Flow diagram of the study design

This study was conducted as part of the Mothers and Children’s Environmental Health (MOCEH) study, which is a hospital- and community-based prospective birth cohort study initiated in 2006 in Korea. The study was approved by the Institutional Review Boards of Ewha Womans University School of Medicine, Dankook University Hospital, and Ulsan University Hospital. Written informed consent was obtained at enrollment from all participants on behalf of themselves and their children. Details of the MOCEH study and its protocols are described elsewhere in a comprehensive review [25]. Briefly, pregnant women in their first trimester were recruited from three university hospitals located in Seoul (metropolitan area), Ulsan (industrial area), and Cheonan (urban area) between August 2006 and October 2011 (*n* = 1,751). Of these, we excluded participants who were pregnant with twins (*n* = 31), who had undergone spontaneous abortions (*n* = 22), with congenital anomalies (*n* = 10), with intra-uterine growth retardation (*n* = 6), with pregnancy complications (hypertension and/or diabetes, *n* = 39), and with preterm delivery (*n* = 59). Of the remaining 1,584 pregnant women, we also excluded those with missing dietary data, and those with implausible energy intakes of < 500 or > 5000 kcal/day (*n* = 237). Lastly, 12-month-old infants who had missing information on AD were also excluded (*n* = 531). Therefore, 816 eligible subjects were included in our final analysis (Fig. 1).

### Maternal UPF consumption

The primary explanatory variable was maternal consumption of UPF. Maternal dietary intake data was assessed by trained dietary interviewers using a single 24-h dietary recall. Participants were asked to complete a dietary survey on all the foods and beverages they had consumed over the last 24 h before the interview. Three days after the interview, all participants received follow-up contact by telephone to ascertain detailed information on the types of foods and drinks consumed, portion size, and extra condiments. Food intake data were analyzed using the computer-aided nutritional analysis program CAN-Pro (version 4.0; Korean Nutrition Society, Seoul, Korea). Food items reported in the 24-h recall were categorized based on the NOVA food classification system for the extent and purpose of industrial processing into unprocessed or minimally processed foods, processed culinary ingredients, processed foods, and UPF [18]. Unprocessed or minimally processed foods include those obtained directly from nature or minimally altered by processes that do not introduce additional ingredients. Processed culinary ingredients consist of products extracted (e.g., plant oils, animal fats, sugar, and starch) from foods or purified substances (e.g., salt) directly from nature. Processed foods are manufactured by adding culinary ingredients to unprocessed or minimally processed foods. This category often involves preservation methods, such as canning, bottling, and fermentation, to enhance storability. UPF are industrial formulations created using substances derived from foods, other organic sources, preservatives, and additives. These foods typically contain little or no original foods and often mimic the appearance, shape, and taste of foods through various processing techniques. The primary goal of these processes is to produce highly convenient, palatable, and profitable products with an extended shelf life. In the 24-h recall dataset, a total of 1,048 food items were consumed either as food or ingredients and classified into one of the four NOVA food groups: unprocessed or minimally processed foods (*n* = 485), processed culinary ingredients (*n* = 27), processed foods (*n* = 130), and UPF (*n* = 406). Then, the percentages of total energy intake derived from the consumption of UPF (%UPF) were computed.

### Assessment of AD occurrence in 12-month-old infants

Mothers/caregivers were queried about infantile symptoms, medical history, and the diagnosis of AD using an International Study of Asthma and Allergies in Childhood (ISAAC)-based questionnaire when the infants reached 12 months of age. ISAAC, founded in 1991, stands as the largest global collaborative research initiative dedicated to investigating asthma, rhinitis, and eczema in children. In cases where an infant exhibited symptoms, visited hospitals, or received a diagnosis of AD, the infant was classified as having an AD [26].

### Determination of the levels of atopy markers in blood

Cord blood (15 mL) was obtained from a cord site close to the infant at birth, using standard commercial evacuated tubes (Vacutainer®, BD, Franklin Lakes, NJ, USA) containing sodium heparin. The collected blood samples were then preserved at −70 °C until analysis. At the Neodin Medical Institute, a laboratory certified by the Korean Ministry of Health and Welfare, the levels of high-sensitivity-C-reactive protein (hs-CRP), interleukin-10 (IL-10), and total immunoglobulin E (total IgE) were assessed as markers of atopy from the cord blood samples.

### Other variables

Trained personnel conducted structured interviews with participants to gather maternal characteristics data, including age, height (m), pre-pregnancy weight (kg), family income (< USD2,000, USD2,000–4,000, > USD4,000), and other relevant information, such as secondhand exposure to cigarette smoke (yes/no) and parental allergic history (yes/no). Pre-pregnancy body mass index (BMI) was computed from the self-reported height and weight information as the ratio of weight (kg) to the square of height (m). Pregnancy outcome data, comprising infant sex, gestational age (days), and birth weight (g), were extracted from medical records. Gestational age at delivery was inferred from the last menstrual period and was also estimated by ultrasonography. Additionally, the duration of breastfeeding up to 12 months was collected from self-reported questionnaires. Maternal blood samples were collected during pregnancy. Blood cadmium [27] was measured by graphite furnace atomic absorption spectrometry with Zeeman background correction (Perkin Elmer AAS800, Perkin Elmer, Waltham, MA, USA). The Korean Healthy Eating Index (KHEI) was used as the diet quality index [28]. The KHEI comprises 14 components in three categories: adequacy, moderation, and energy balance. Eight components—whole grains, refined grains, fruit intake (excluding juice), fruit intake (including juice), vegetable intake (excluding kimchi and pickles), vegetable intake (including kimchi or pickles), the ratio of white meat to red meat, and the percentage of energy from carbohydrates—are worth 0–5 points each. Six components—breakfast consumption, milk and dairy intake, protein foods, sodium intake, the percentage of energy from empty calorie foods, and the percentage of energy from fat—are worth 0–10 points each. The total KHEI scores thus range from 0 to 100, with a higher score indicating a better quality diet that meets the Korean guidelines for a healthy diet [28].

### Statistical analysis

Descriptive statistics were used to examine the frequencies and means ± standard deviations (SDs) of sociodemographic status and lifestyle variables. The participants were categorized into quartiles based on the percentage of energy contributed by UPF: Q1 (< 6.7%), Q2 (6.7–10.5%), Q3 (10.6–15.5%), and Q4 (15.5–58.9%). Distributions of sociodemographic status, lifestyle variables, energy and nutrient intakes, proportions of acceptable macronutrient distribution range (AMDR), and biochemical marker levels were examined in relation to the quartiles of maternal UPF consumption. The chi-square test for categorical variables was used to examine the differences across the quartiles of the UPF consumption. One-way analysis of variance (ANOVA), followed by Tukey’s post hoc test for continuous variables, was conducted to identify any significant differences across the quartiles of the UPF consumption. We calculated the least square means (LS-means) and standard deviations using generalized linear models to examine the associations of each atopy marker in cord blood with the quartiles of the UPF consumption. *P*-values for trends were assessed by modeling the median value of the quartiles in the linear regression analysis. Multiple logistic regression was used to calculate adjusted odds ratios (ORs) with 95% confidence intervals (CIs) to examine the relationship between the quartiles of maternal UPF consumption and the risk of infantile AD. We adjusted for maternal age, pre-pregnancy BMI, family income level, parity, indirect smoking, maternal energy intake (log-transformed), infant’s sex, birth weight, blood cadmium at pregnancy (log-transformed), parental allergic history, and the KHEI. A complete case analysis was performed to remove cases with missing values and ensure that we analyzed only complete cases. All statistical analyses were performed using SAS (version 9.4; SAS Institute, Cary, NC, USA), and results were regarded as significant at *P* < 0.05.

## Results

### Characteristics of the study participants

Table 1 summarizes the characteristics of pregnant women and their babies by quartiles of maternal UPF consumption. Significant differences in household income level, parity, and infant birth weight (all *P* < 0.05) were observed among the subjects based on maternal consumption of UPF.

**Table 1:** General characteristics of study participants by quartiles of maternal percentage energy intake from UPF consumption

Characteristic	*n*	Total	Quartiles of UPF consumption, ^1^%TE				*P*-value
			Q1	Q2	Q3	Q4	
*UPF consumption, %TE*							
Range	816	0.0–58.9	0.0–6.8	6.8–10.6	10.6–15.5	15.5–58.9	
Mean ± standard deviation	816	12.4 ± 8.5	4.5 ± 1.7	8.7 ± 1.1	12.9 ± 1.4	23.6 ± 8.9	< 0.0001
*Maternal characteristics*							
Age, years	816	30.1 ± 3.5	30.1 ± 3.6	30.1 ± 3.6	30.5 ± 3.5	29.7 ± 3.4	0.1408
Pre-pregnancy BMI, kg/m^2^	800	21.1 ± 2.9	21.5 ± 2.9	20.8 ± 2.7	21.2 ± 3.4	21.1 ± 2.7	0.1392
Income (million KRW/month)	786						
< 2		215(27.4)	71(36.1)	51(25.6)	47(23.9)	47(24.1)	0.0039
2−3		284(36.1)	70(36.1)	66(33.2)	64(32.5)	83(42.6)	
≥ 3		287(36.6)	54(27.8)	82(41.2)	86(43.7)	65(33.3)	
Parity	707						0.0234
Nulliparous		360(51.0)	77(44.4)	94(53.1)	83(47.4)	106(59.2)	
Multiparous		347(49.0)	97(55.6)	85(46.9)	92(52.6)	73(40.8)	
Passive smoking status	816						0.6244
Never		327(40.1)	89(43.4)	84(41.2)	77(37.8)	78(38.2)	
Ever		489(59.9)	115(56.7)	120(58.8)	127(62.3)	126(61.8)	
Parental allergic disease history	816						0.8890
No		437(53.6)	107(52.4)	108(52.9)	114(55.9)	107(52.5)	
Yes		379(46.4)	97(47.6)	96(47.1)	90(44.1)	97(47.6)	
Blood cadmium level, µg/L	730	1.5 ± 0.6	1.5 ± 0.8	1.4 ± 0.4	1.5 ± 0.7	1.4 ± 0.4	0.3824
*Babies characteristics*							
Sex	816						0.8376
Boys		424(52.0)	107(52.7)	106(52.0)	110(53.9)	101(49.5)	
Girls		392(48.0)	97(47.3)	98(48.0)	94(46.1)	103(50.5)	
Birth weight, g	816	3299.3 ± 385.4	3343.3 ± 395.7	3250.5 ± 362.4	3332.8 ± 407.7	3270.7 ± 368.6	0.0349
Duration of breastfeeding, month	777	8.4 ± 4.4	8.4 ± 4.4	8.3 ± 4.5	8.2 ± 4.6	8.9 ± 4.2	0.4608

All values are number (%) or mean ± SD.

BMI, body mass index; TE, percentage of total energy intake; UPF, ultra-processed food

*P*-values for chi-square test or analysis of variance

### Energy and nutrient intake according to quartiles of maternal UPF intake

Energy and nutrient intakes by quartiles of maternal UPF consumption during pregnancy are presented (Table 2). In relation to the daily energy intake of Korean adults (1,826 kcal), 61.8% was derived from carbohydrates, 15.5% from proteins, and 23.6% from fat. The daily energy intake and the energy contribution of total fat were significantly higher in Q4, characterized by a high UPF energy contribution, compared to Q1, which had a low UPF energy contribution (1,693.7 kcal, 21.2% in Q1 versus 1,942.3 kcal, 25.6% in Q4, *P* < 0.0001). Conversely, the energy contribution from protein was significantly lower in Q4 (14.6%) compared to Q1 (16.3%; *P* < 0.0001). Additionally, there were significant differences in the intakes of vegetables and dietary fiber (302.7 and 21.9 g in Q1 versus 231.1 and 19.3 g in Q4, *P* < 0.005). The intake of vitamin E, an antioxidant nutrient, was also significantly lower in Q4, with a high UPF energy contribution compared to other groups (*P* = 0.0360).

**Table 2:** Nutrient intakes of the study participants according to quartiles of maternal percentage energy intake from UPF consumption

Characteristic	Total	Quartiles of UPF consumption				*P*-value
		Q1	Q2	Q3	Q4	
Range of UPF consumption, %TE		0.0–6.8	6.8–10.6	10.6–15.5	15.5–58.9	
Total energy, kcal	1826.1 ± 491.5	1693.7 ± 455.0^a^	1826.3 ± 507.1^b^	1841.4 ± 470.7^b^	1942.3 ± 502.5^b^	< 0.0001
Carbohydrate, %TE	61.8 ± 9.7	63.2 ± 9.3	61.2 ± 10.0	61.4 ± 9.8	61.2 ± 9.8	0.0994
Protein, %TE	15.5 ± 3.5	16.3 ± 3.9^a^	16.0 ± 3.4^a^	15.1 ± 2.9^b^	14.6 ± 3.3^b^	< 0.0001
Fat, %TE	23.6 ± 7.7	21.2 ± 7.1^a^	23.4 ± 7.7^b^	24.2 ± 7.9^bc^	25.6 ± 7.4^c^	< 0.0001
Cholesterol, mg	305.4 ± 194.4	287.0 ± 199.6	330.6 ± 206.7	305.9 ± 184.0	298.0 ± 185.1	0.1353
Fiber, g	20.7 ± 7.6	21.9 ± 7.8^a^	21.2 ± 7.3 ^ab^	20.5 ± 7.9^ab^	19.3 ± 7.0^b^	0.0047
Sodium, g	4106.2 ± 1592.6	4009.0 ± 1536.8	4278.8 ± 1748.2	4213.5 ± 1595.2	3923.0 ± 7.0	0.0791
Vitamin A, µg RE	754.8 ± 538.1	746.3 ± 562.1	753.3 ± 413.0	789.01 ± 672.6	730.3 ± 471.1	0.7299
Vitamin C, mg	141.4 ± 110.8	154.8 ± 119.9	138.6 ± 110.5	128.6 ± 110.2	143.6 ± 101.0	0.1155
Vitamin E, mg	16.4 ± 8.3	16.5 ± 7.1^ab^	17.6 ± 9.2 ^a^	16.4 ± 8.1^ab^	15.2 ± 8.6^b^	0.0360
Vegetables, g	269.1 ± 164.5	302.7 ± 170.8^a^	281.5 ± 154.7^a^	261.3 ± 163.3^ab^	231.1 ± 161.6^b^	< 0.0001
Fruits, g	344.8 ± 347.7	355.7 ± 367.4	337.0 ± 349.4	324.9 ± 353.1	362.1 ± 320.8	0.6925
KHEI	57.4 ± 0.7	58.1 ± 0.7	57.6 ± 0.7	57.0 ± 0.7	56.9 ± 0.7	0.6135

All values are mean ± SD.

Values followed by different superscript letters (a, b, c) are significantly different by Tukey’s test (*P* < 0.05)

TE, percentage of total energy intake; UPF, ultra-processed food; KHEI, the Korean Healthy Eating Index score

### Atopic marker levels according to quartiles of maternal UPF intake

Atopic marker levels across the quartiles of maternal UPF intake are shown in Table 3. The levels of hs-CRP from the cord blood increased significantly from Q1 to Q4 (*P* for trend < 0.05) for the maternal UPF intake. However, no linear association was observed between maternal UPF intake and levels of IL-10 and total IgE.

**Table 3:** Infant’s CRP, IL-10, and IgE according to quartiles of maternal percentage energy intake from UPF consumption

	Q1	Q2	Q3	Q4	*P* for trend
CRP	0.018 ± 0.007	0.018 ± 0.007	0.022 ± 0.008	0.037 ± 0.007	0.0493
lL-10	3.770 ± 2.052	2.440 ± 2.008	4.190 ± 2.007	1.977 ± 1.802	0.4255
IgE	2.155 ± 2.554	5.794 ± 2.453	2.200 ± 2.545	7.279 ± 2.390	0.0682

All values are LS-mean ± SD.

*P* for the linear trends by quartiles of sex-specific quartiles of UPF consumption (percentage of total energy intake, %TE).

Adjusted for maternal age, pre-pregnancy body mass index (BMI), parity, family income level, indirect smocking, maternal energy intake (log-transformed), infant’s sex, birth weight, blood cadmium at pregnancy (log-transformed), and parental allergic history

UPF, ultra-processed food

### Association between maternal UPF intake and infantile AD

Table 4 presents OR and 95% CI for the risk of infantile AD within 12 months. In the unadjusted logistic analysis, there was a tendency for an exposure−response relationship between the maternal UPF intake and the risk of infantile AD within 12 months (*P* for trend = 0.0436). The crude ORs in Q1, Q2, Q3, and Q4 were 1.00 (reference), 1.45 (95% CI: 0.93–2.31), 1.35 (95% CI: 0.85–2.15), and 1.69 (95% CI: 1.07–2.66), respectively. These relationships became more statistically significant after adjusting for confounding factors (*P* for trend = 0.0418): the adjusted ORs for Q1, Q2, Q3, and Q4 were 1.00 (reference), 1.69 (95% CI: 0.86–3.34), 1.95 (95% CI: 0.98–3.88), and 2.19 (95% CI: 1.11–4.32). There was a significantly greater risk of developing AD within 12 months for children born to mothers in the highest quartile of UPF than for children born to mothers in the lowest quartile, even after adjusting for the KHEI [odds ratio (OR) = 1.805; 95% confidence interval (CI): 1.018–3.203].

**Table 4:** Odds ratios with 95% confidence intervals of the quartiles of maternal percentage energy intake from UPF consumption and atopy in 12-month-old infants

	Q1	Q2	Q3	Q4	*P* for trend
Range of UPF consumption, %TE	0.0–6.8	6.8–10.6	10.6–15.5	15.5–58.9	
Cases, *n*	41(20.1)	55(27.0)	53(25.9)	61(30.0)	
Model 1	1(ref)	1.467(0.925−2.314)	1.352(0.849−2.152)	1.685(1.069−2.657)	0.0436
Model 2	1(ref)	1.694(0.858−3.344)	1.949(0.979−3.881)	2.189(1.110−4.317)	0.0418
Model 3	1(ref)	1.625(0.921−2.865)	1.632(0.920−2.892)	1.805(1.018−3.203)	0.0541

Model 1: Unadjusted

Model 2: Adjusted for maternal age, pre-pregnancy body mass index (BMI), parity, income level, indirect smocking, maternal energy intake (log-transformed), blood cadmium at pregnancy (log-transformed), infant’s sex, birth weight, breastfeeding duration, and parental allergic history

Model 3: Adjusted for the adjustment variables in model 2 along with the Korean Healthy Eating Index (KHEI).

TE, percentage of total energy intake; UPF, ultra-processed food

## Discussion

In this prospective cohort study of mothers and children, our findings indicate that maternal consumption of UPF during pregnancy is associated with an increased risk of infantile AD within the first year of life. Infants born to mothers in the highest consumption group of UPF had more than twice the risk of developing AD compared to those born to mothers in the lowest consumption group, and this association was maintained even after the additional adjustment for the KHEI. Our study is the first to examine the associations between maternal UPF intake and infantile AD.

In our study, the average contribution of UPF to total dietary energy intake in the population was 12.4%. This result was relatively lower than the results reported in a Western study focusing on pregnant women, which indicated a range of 15.2−54.4% [22, 24, 29]. Brazilian pregnant women obtained 15.2% of their energy intake from UPF [29], while the corresponding figures for Norway [24] and the United States [22] were reported as 31.8% and 54.4%, respectively. Consistent with our study findings, a previous study conducted on the general Korean population [30] also reported a lower contribution of UPF to average dietary energy intake (17.8%) compared to Western nations. Discrepancies in the proportion of energy contributed by UPF across studies may be partially attributed to differences in dietary data collection tools, such as 24-h dietary recall, food frequency questionnaires, and the classification of food groups using the NOVA system. Additionally, variations in dietary culture may offer a partial explanation for the observed differences.

Previous studies have explored the impact of mothers’ consumption of UPF during pregnancy on the health of their children [22, 23]. For instance, Rohatgi et al. discovered that an increase in the dietary share of UPF during pregnancy was linked to an increase in thigh skin wrinkles, subscapular skin wrinkles, and overall body fat [22]. Their study provided evidence that increased consumption of UPF is associated with a higher incidence of adverse pregnancy outcomes, including excessive weight gain during pregnancy and increased neonatal body fat. Ben-Avraham et al. found that maternal UPF intake was linked to maternal obesity and a shorter male infant anogenital distance, indicating potential adverse effects on both maternal and neonatal health [31].

Many prior transgenerational investigations have primarily examined the association between the overall quality of maternal diet and infantile AD, suggesting that adhering to a healthier dietary pattern during pregnancy could be linked to a reduced risk of infantile AD (8−17). In a Canadian cohort study, the adoption of a plant-based diet evaluated at 24–28 weeks of gestation was linked to a decreased likelihood of infantile AD at 1 year (OR = 0.65; 95% CI: 0.56−0.75) [15]. Similarly, a prospective cohort study conducted in Guangzhou, a city in southern China, revealed that both the plant pattern and the dairy and eggs pattern during pregnancy (assessed at 20–28 weeks of gestation) were associated with a reduced risk of infantile AD at 6 months [16]. However, these dietary patterns cannot often discern the extent of industrial modifications within foods belonging to the same group (e.g., fresh fruit versus sweetened fruit juice). In contrast, our study, using the NOVA classification system to differentiate UPF from others [18], provides robust epidemiological evidence regarding the impact of maternal UPF consumption on the development of infantile AD. Furthermore, our findings may support more actionable and specific dietary guidance to decrease UPF intake for mitigating the risk of AD in comparison to broader recommendations advocating for a generally healthier diet.

A high energy contribution of UPF is negatively related to overall diet quality [32, 33]. In the current study, we also found that the total energy intake and energy contribution rate of fat tended to increase with the UPF intake quartile. Conversely, the intake of vegetables, dietary fiber, and antioxidant nutrients, such as vitamin E, tended to decrease. This raises the question of whether the significant relationship between UPF intake and AD prevalence is due to the poor nutritional quality associated with UPF or if the UPF itself has harmful effects [34, 35]. In many epidemiological studies finding a significant association between UPF and health-related outcomes, diet quality adjustments did not alter the significance of the association [30, 34, 35]. Similarly, our study also retained the significant association between UPF and AD outcomes after adjusting for the KHEI. Indeed, the dietary quality adjustment had a minimal impact. Further research is required to determine whether dietary quality mediates the relationship between UPF intake and AD.

In our study, there was no positive association between the dietary intake of sodium and UPF observed in Western countries, such as the United Kingdom [36] and Australia [37]. However, this finding was similar to research conducted in South Korea [30, 38] that observed a decrease in the dietary intake of sodium with increasing consumption of UPF. These discrepancies are thought to be due to different major sodium sources across countries. The major contributing dietary sodium source in South Korea is kimchi [39], which was not classified as UPF in our study.

The mechanisms underlying the relationship between the consumption of UPF and the onset of AD conditions remain complex and multifaceted. One potential contributor is the intricate interplay between dietary factors and immune system modulation. Processed foods, often rich in additives, preservatives, and artificial ingredients, may influence the gut microbiota composition and function, impacting immune responses [40]. Additionally, the high levels of sugar and unhealthy fats commonly found in processed foods may contribute to systemic inflammation [41], which has been implicated in the development of AD [42]. Furthermore, the processing methods themselves, involving high temperatures and pressure, could lead to the formation of advanced glycation end products, which might influence immune reactivity [43]. Overall, the intricate connections between processed food consumption and AD manifestations involve a combination of immunological, inflammatory, and metabolic factors that warrant further exploration. Due to the absence of infant blood collection in this study, our attempt to investigate the correlation between maternal UPF intake and infant’s inflammatory biomarkers encountered limitations. Nevertheless, upon analyzing the correlation using blood obtained from umbilical cord samples, no observable correlation was found between IL-10 and total IgE, whereas the inflammatory marker hs-CRP exhibited a significant positive correlation with UPF intake. Further investigations, encompassing a broader array of immune cytokines, are essential to unravel the mechanisms underpinning the connection between maternal UPF intake and the development of AD in infants.

Our study had several limitations. Firstly, the study population was relatively small compared to other cohort studies, although we emphasize the meticulously controlled prospective birth cohort design of the MOCEH study in Korea. Secondly, the identification of AD relied on parental reporting, introducing the potential for a lack of standardization, misclassification, or recall bias, with the chance of over-reporting. Lastly, despite adjustments for certain variables as potential confounding factors, our findings could still be affected by residual and unmeasured confounding. Also, we could not conduct analyses based on diet assessment time, despite its long observational period (12–28 weeks). The period from 12 to 28 weeks corresponds to the second trimester of pregnancy, and, as this timeframe is typically regarded as a stable period in terms of nutritional needs and dietary recommendations, assessment time was not considered [44]. Additional limitations include the estimation of UPF intake through the 24-h recall method. A single 24-h recall is not considered representative of an individual’s habitual diet. To accurately assess an individual’s habitual diet, repeated 24-h dietary recalls are recommended [45]. Nevertheless, we regard this study as the first in Koreans to investigate the relationship between maternal UPF intake and infantile AD.

## Conclusions

In conclusion, we observed that higher maternal consumption of UPF during pregnancy was associated with a greater risk of infantile AD within the first year of life in this cohort study. These findings offer insights into potential avenues for the prevention of infantile AD. Further studies are necessary to confirm and gain a deeper understanding of this association.

## Data Availability

No datasets were generated or analysed during the current study.

## Abbreviations

AD: Atopic dermatitis
BMI: Body max index
hs-CRP: High-sensitivity-C-reactive protein
IL-10: Interleukin-10
ISAAC: International Study of Asthma and Allergies in Childhood
MOCEH: Mothers and Children’s Environmental Health (MOCEH) study
total IgE: Total immunoglobulin E
UPF: Ultra-processed food
